# Around the Hospitals

**Published:** 1893-04-01

**Authors:** 


### Around the Hospitals.

**The Royal Hospital for Diseases of the Chest** held its annual meeting on the 15th inst. The Lord Mayor, who presided, was supported by Lord Charles Bruce, the president of the institution, and many other influential gentlemen. The report for the past year shows that 542 in-patients and 6,231 out-patients have been relieved. Progress has been made by the reduction of expenditure, and an improvement in the domestic arrangements through the employment of more women and fewer men servants, which as resulted in greater economy, efficiency, and cleanliness; whilst donations show a slight falling off, annual subscriptions show a marked increase. The hospital contains several empty wards, for which needy sufferers are ready were funds forthcoming to render it possible to admit them.

**The Metropolitan Hospital** held its annual meeting on Friday last, Mr. C. J. Thomas, C.C., presiding. The report of the committee, which was submitted by the secretary (Mr. Charles H. Byers), showed that the work of the hospital had largely increased last year, the number of in-patients having risen from 880 to 887, and the out-patient attendances from 69,196 to 71,754. The receipts amounted to £7,004, and the expenditure to £8,885, and the adverse balance, which at the commencement of the year was £3,651, had increased to £5,531. Under these circumstances the committee had been forced to close twenty-four beds, so that at the present time only fifty-four were available for the reception of patients. The Duke of Portland had consented to preside at the festival dinner to be held in May, when it is trusted that sufficient funds will be forthcoming to enable the committee to at least reopen the ward just closed. The Chairman made a very urgent appeal for increased public support to an institution which, he said, was doing so much good to the poor of the neighbourhood. He regretted that the committee had had to borrow another £4,000 on mortgage in order to pay the tradesmen's bills as far as possible up to the end of the year, thus raising the mortgage debt to £10,000. He pointed out that the bequest of the late Mr. Henry Spicer, which would probably amount to about £11,500, could not be used for the general purposes of the hospital, but to be invested, and the interest derived therefrom used solely for the benefit of convalescent patients.

**The Birmingham General Hospital** has now passed its 113th anniversary. The progress of the past year has been very satisfactory. The average daily number of in-patients was nearly three times the number received in 1891. Financially, progress has also been made, and during the past year the institution has been fortunate as to legacies. Annual subscriptions will be much needed when the new hospital is occupied, as a larger number of patients will have to be provided for. It is a distinct feature of improvement of the year that patients are now provided with tea and sugar by the hospital. The contributions towards the new building have reached the sum of £93,032.

